# Comparative analysis of histologically classified oligodendrogliomas reveals characteristic molecular differences between subgroups

**DOI:** 10.1186/s12885-018-4251-7

**Published:** 2018-04-10

**Authors:** Chris Lauber, Barbara Klink, Michael Seifert

**Affiliations:** 1 Institute for Medical Informatics and Biometry, Carl Gustav Carus Faculty of Medicine, Technische Universität Dresden, Dresden, Germany; 2 Institute for Clinical Genetics, Carl Gustav Carus Faculty of Medicine, Technische Universität Dresden, Dresden, Germany; 3 National Center for Tumor Diseases, Dresden, Germany

**Keywords:** Histologically classified oligodendrogliomas, Molecular subgroup, Gene expression signature, Gene regulatory network, Bioinformatics, Computational systems biology

## Abstract

**Background:**

Molecular data of histologically classified oligodendrogliomas are available offering the possibility to stratify these human brain tumors into clinically relevant molecular subtypes.

**Methods:**

Gene copy number, mutation, and expression data of 193 histologically classified oligodendrogliomas from The Cancer Genome Atlas (TCGA) were analyzed by well-established computational approaches (unsupervised clustering, statistical testing, network inference).

**Results:**

We applied hierarchical clustering to tumor gene copy number profiles and revealed three molecular subgroups within histologically classified oligodendrogliomas. We further screened these subgroups for molecular glioma markers (1p/19q co-deletion, *IDH* mutation, gain of chromosome 7 and loss of chromosome 10) and found that our subgroups largely resemble known molecular glioma subtypes. We excluded glioblastoma-like tumors (7a10d subgroup) and derived a gene expression signature distinguishing histologically classified oligodendrogliomas with concurrent 1p/19q co-deletion and *IDH* mutation (1p/19q subgroup) from those with predominant *IDH* mutation alone (IDHme subgroup). Interestingly, many signature genes were part of signaling pathways involved in the regulation of cell proliferation, differentiation, migration, and cell-cell contacts. We further learned a gene regulatory network associated with the gene expression signature revealing novel putative major regulators with functions in cytoskeleton remodeling (e.g. *APBB1IP*, *VAV1*, *ARPC1B*), apoptosis (*CCNL2*, *CREB3L1*), and neural development (e.g. *MYTIL*, *SCRT1*, *MEF2C*) potentially contributing to the manifestation of differences between both subgroups. Moreover, we revealed characteristic expression differences of several *HOX* and *SOX* transcription factors suggesting the activity of different glioma stemness programs in both subgroups.

**Conclusions:**

We show that gene copy number profiles alone are sufficient to derive molecular subgroups of histologically classified oligodendrogliomas that are well-embedded into general glioma classification schemes. Moreover, our revealed novel putative major regulators and characteristic stemness signatures indicate that different developmental programs might be active in these subgroups, providing a basis for future studies.

**Electronic supplementary material:**

The online version of this article (10.1186/s12885-018-4251-7) contains supplementary material, which is available to authorized users.

## Background

Oligodendrogliomas belong to the class of diffuse gliomas that represent the most frequent primary brain tumors in adults [1]. About 4 to 8% of all diagnosed tumors of the central nervous system are oligodendrogliomas [2]. Diffuse gliomas are generally characterized by infiltration of the surrounding brain tissue, and fast progression and relapse are common [3]. Traditionally, histological similarities to normal glial cells (astrocytes and oligodendrocytes) were used to distinguish between different types of diffuse gliomas according to the World Health Organization (WHO) 2007 grading system [4]. Known downsides of this histological classification include a considerable variability of diagnoses between neuropathologists and difficulties in discriminating oligodendrogliomas from other types of diffuse gliomas like astrocytomas and “mixed-type” oligoastrocytomas, which complicates diagnostics and treatment decisions for individual patients [5, 6]. These challenges led to the exploration of molecular markers for glioma diagnostics [7]. The majority of oligodendrogliomas shows a characteristic allelic loss of chromosomal arms 1p and 19q (1p/19q) that contributes to better chemotherapy sensitivity and longer recurrence-free survival [8, 9]. Three different gene expression subtypes of 1p/19q co-deleted oligodendrogliomas have recently been revealed, but the analysis of the clinical relevance of these subtypes requires additional studies [10]. Further, specific heterozygous somatic point mutations of the isocitrate dehydrogenase gene (IDH1/2) were found in more than three-fourths of all oligodendrogliomas and nearly three-fourths of all astrocytomas of WHO grades II and III [11–13] and in all 1p/19q codeleted gliomas [14]. These mutations are associated with the glioma-CpG island methylator phenotype (G-CIMP) [15, 16] and with a better prognosis compared to IDH wild-type tumors [11, 17].

These molecular markers were integrated into a recent update of the classification of tumors of the central nervous system by the WHO [18]. As a consequence, some diffuse glioma classes became obsolete, like the “mixed-type” oligoastrocytomas that should now be classified as either oligodendrogliomas or astrocytomas. According to this new classification, oligodendrogliomas are characterized by the co-occurrence of the mutation of *IDH1/2* and the 1p/19q co-deletion. Notably, this class does not accommodate *IDH*-mutated tumors with 1p/19q wild-type that were classified as oligodendrogliomas based on histology before. Such discrepancies between histological and molecular tumor classification still remain a great challenge for further improvements of glioma diagnostics, but in terms of prognosis molecular markers can outweigh histological characteristics. Recently, it has been shown that glioma subgroups can be defined based on *IDH* mutation and 1p/19q co-deletion status deriving genetic subgroups that are more reflective of disease subtypes than glioma classes defined by histology [19]. These results were further refined through the analysis of DNA methylation profiles revealing clinically relevant molecular subtypes [20]. In addition, single cell transcriptome data has allowed to gain novel insights into the molecular architecture of oligodendrogliomas showing that the majority of tumor cells express either a specialized astrocyte-like or oligodendrocyte-like program, whereas a subpopulation of cells remains undifferentiated and is associated with a neural stem cell expression program that most likely drives tumor development [21]. This has been further extended by analyzing single cell transcriptomes of oligodendrogliomas and astrocytomas suggesting a common stemness program for both tumor types that drives tumor growth, whereas differences between both types are mainly driven by the tumor microenvironemt and specific genetic signatures [22]. This has important consequences for the clinical management of oligodendrogliomas and may also explain in part differences between molecular and histological classifications. All these and many other studies have greatly contributed to a better understanding of molecular characteristics of oligodendrogliomas. Still, also in the light of differences between histological and molecular classifications, our knowledge about specific molecular characteristics of oligodendrogliomas is incomplete.

Here, we present an in-depth computational analysis of histologically classified oligodendrogliomas from The Cancer Genome Atlas (TCGA) revealing novel differences between molecular subgroups at the level of individual genes, pathways, and gene regulatory networks. We first stratified these tumors based on their gene copy number profiles into three subgroups utilizing unsupervised clustering. Additional screening for the presence of known glioma markers showed that these subgroups largely resembled already known molecular glioma subtypes. To further characterize molecular differences, we derived a signature of differentially expressed genes distinguishing tumors with 1p/19 co-deletion and *IDH* mutation from tumors that predominantly showed an *IDH* mutation. We further learned a gene regulatory network that is capable to explain this observed expression signature. This enabled us to identify novel putative major regulators that are potentially involved in the manifestation of differences between both subgroups. Interestingly, this network also contained a characteristic expression signature of *HOX* and *SOX* genes that distinguishes both subgroups indicating the activity of different glioma stemness programs.

## Methods

### Molecular data of oligodendrogliomas and normal brains

DNA copy number, RNA-seq gene expression, and somatic mutation data was obtained from the TCGA data portal (https://gdc.cancer.gov/) for 193 histologically classified oligodendrogliomas of the TCGA lower grade glioma (LGG) cohort (Additional file 1). The vast majority of tumor samples represented primary tumors, except five recurrent tumors. We determined gene-specific copy number log-ratios for each oligodendroglioma based on its corresponding DNA copy number profile (see [23] for details). Three commercially available normal brain samples were obtained from StrataGen, BioChain, and Clonetech for which RNA-seq gene expression has been measured previously. All considered gene copy (17,677 genes) and gene expression (15,988 genes) profiles are provided in Additional file 2.

### Clustering based on CNV data

Hierarchical clustering (euclidean distance, complete linkage) of tumors was done in R using the processed gene copy number variation (CNV) log-ratio data of tumor compared to normal. One obvious outlier (TCGA-P5-A5F6-01A) was removed from subsequent analyses. Three tumor subgroups were derived by cutting the clustering dendrogram into three sub-trees. These subgroups were named taking into account the following molecular properties: (i) 1p/19q - co-deletion of chromosomal arms 1p and 19q and presence of characteristic *IDH1/2* mutation, (ii) IDHme - predominance of *IDH1/2* mutation but no co-deletion of 1p and 19q, and (iii) 7a10d - no co-deletion of 1p and 19q, lack of *IDH1/2* mutations, amplification of chromosome 7, and deletion of chromosome 10.

### Data normalization and identification of differentially expressed genes

Raw RNA-seq gene expression counts were loaded into R. Combined normalization of tumor and normal brain RNA-seq data was done using the voom function of the limma package [24] with normalization method cyclic loess. Differential gene expression analysis between CNV-derived tumor subgroups was done following limma’s standard workflow. Differentially expressed (signature) genes were selected using an FDR-adjusted *p*-value (*q*-value) [25] cut-off of 0.01.

### Verhaak and G-CIMP classification

Gene expression log_2_-ratios of genes in tumor compared to the average expression in normal brain tissue were computed for each oligodendroglioma sample. 756 of 840 genes that were used to derive the four Verhaak classes [26] were part of our data set. We calculated pearson correlation and associated p-values between the gene expression log-ratios in the glioma reference set and our tumor subgroups. Similarly, 42 of 50 genes of the glioma-CpG island methylator phenotype (G-CIMP) set [15] were part of our data set, for which we calculated Pearson correlations and *p*-values. Note that genes missing from the Verhaak and G-CIMP signature do not strongly affect the classification, because there are other genes in these signatures that show expression levels that are strongly correlated with those of the missing genes [27].

### Survival analysis

Information about days to death or days to last follow-up was taken from Table S1 of [20]. This table represented the most recent survival information in months at the time of our study. We transformed the survival information from months into days using the factor 30.4167 followed by a rounding to the nearest integer (Additional file 1). We generated survival curves and performed log-rank tests using the R package survival [28].

### Gene and pathway annotation enrichment analysis

Gene, signaling, and metabolome pathway annotations were obtained from [23]. The number of signature genes per annotation category was counted separately for up- and downregulated genes, and the significance of gene enrichment was calculated using Fisher’s exact test.

### Signature-specific regulatory network inference

We inferred transcriptional regulatory networks associated with the normalized expression of the signature genes that discriminate between the 1p/19q and IDHme subgroups following the approach detailed in [27] with few modifications. We constructed two types of networks that differed in the set of predictor variables: (i) only the gene copy number of a signature gene was used to predict its own expression and (ii) in addition to the copy numbers, the gene expression of all signature genes that were annotated as transcription factors (TFs) were used to predict the expression of a signature gene. The expression value of a particular TF was excluded from its own prediction in the latter analysis. For each signature gene, lasso (least absolute shrinkage and selection operator) regression [29] and a significance test for lasso [30] were used to estimate the coefficients and their corresponding significance for each predictor of the underlying signature gene-specific linear model as implemented in [31]. We only considered the most significant predictors with p-values less than 5×10^−5^ specified by the standard detection limit of the covariance test implementation [30]. We further validated each network through cross-validation by repeated random subsampling. To this end, the data was randomly partitioned into a training set constituting two-third of the tumors on which the network was constructed and a test set constituting the remaining one-third of tumors for which the expression of the signature genes was predicted and compared to the experimentally measured expression. This was repeated 100 times. To assess prediction accuracy we calculated pearson correlation of predicted and measured gene expression averaged over the 100 networks. For network visualization we only kept links that occurred in at least 75% of the 100 networks.

## Results

### Gene copy number variations and *IDH* mutations characterize three molecular subgroups of histologically classified oligodendrogliomas

**Fig. 1:**
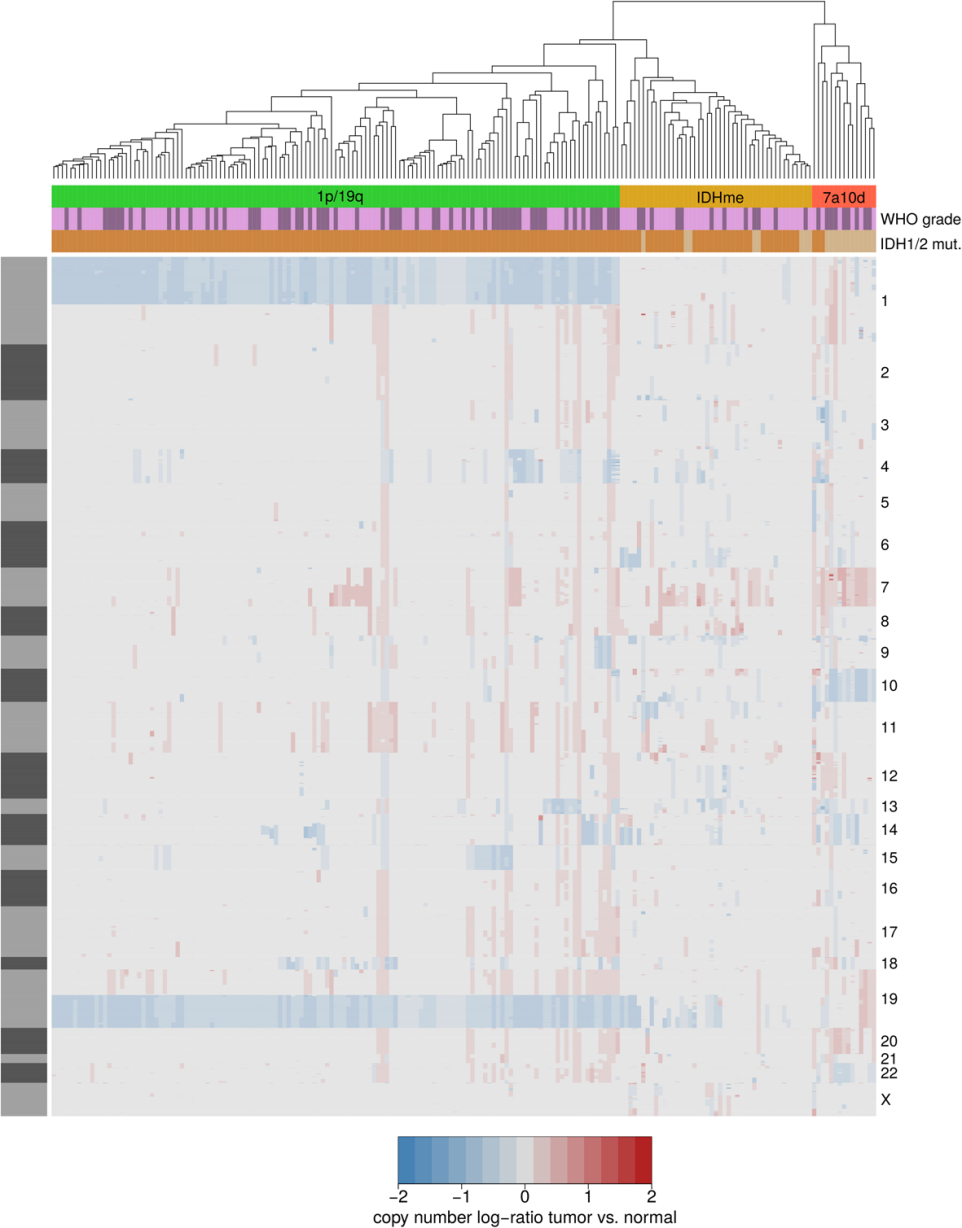
CNV-derived molecular subgroups of histologically classified oligodendrogliomas. Heatmap of genome-wide gene copy number log-ratios of 193 histologically classified oligodendrogliomas (columns) compared to normal brain for 17677 genes (rows); blue: deletions, gray: unchanged, red: amplifications. Chromosomes are highlighted by alternating gray and black bars to the left; chromosome midpoints are indicated by labels to the right. Oligodendroglioma subgroups were revealed by unsupervised clustering and are shown using green (1p/19q), yellow (IDHme), and red (7a10d) column coloring. Tumors of WHO grade II are labeled in light purple. WHO grade III is labeled in dark purple. Tumors with an IDH1/2 mutation are labeled in dark brown. The absence of an IDH mutation is labeled in light brown

It has been shown previously that the majority of histologically classified oligodendrogliomas has a co-deletion of chromosomal arms 1p and 19q and a characteristic mutation of *IDH1/2* [19, 32]. We thus analyzed genome-wide gene copy number data that were available for 193 histologically classified oligodendrogliomas from TCGA (Additional files 1 and 2). Unsupervised clustering of the tumors based on their CNV profiles alone revealed three subgroups (Fig. 1). We further analyzed the mutation status of *IDH1/2* of tumors in these subgroups (Table 1). The largest subgroup comprised 133 tumors (68.9%) and showed the characteristic 1p/19q co-deletion as well as *IDH1* or *IDH2* mutations in each tumor. We refer to this subgroup as 1p/19q. The second largest subgroup included 45 tumors (23.3%) that showed no obvious pattern of gene deletions or amplifications. Since the majority of tumors in this subgroup had an *IDH1/2* mutation (82%), we named this subgroup IDH mutation-enriched (IDHme). The third subgroup comprised 15 tumors (7.8%) that were characterized by an amplification of chromosome 7 and a deletion of chromosome 10 as typically observed in classical glioblastomas [3]. Only three tumors in this subgroup had an *IDH1* or *IDH2* mutation (20%). We refer to this subgroup as 7a10d. It is important to note that the 7a10d subgroup formed an own subcluster that is relatively distant to the 1p/19q and IDHme subgroups, which were both part of one larger subcluster (Fig. 1).

**Table 1:** Frequency of mutations of known cancer-relevant genes per oligodendroglioma subgroup

Gene	Mutated	1p/19q	IDHme	7a10d
*IDH1/2*	Yes	133	37	3
	No	0	8	12
*TP53*	Yes	7	35	6
	No	126	10	9
*ATRX*	Yes	3	30	3
	No	130	15	12
*CIC*	Yes	85	2	0
	No	48	43	15
*FUBP1*	Yes	38	0	0
	No	95	45	15
*NOTCH1*	Yes	31	3	0
	No	102	42	15

### Tumors of the three subgroups differ in mutational status of other cancer-relevant genes

We further observed differences in mutational profiles of known glioma-relevant genes (*TP53*, *ATRX*, *CIC*, *FUBP1*, *NOTCH1* [3, 19]) between tumors of the three subgroups (Table 1). Only 5% and 2% of the 1p/19q tumors showed a mutation of, respectively, *TP53* and *ATRX*, while about two-third of the IDHme tumors had at least one of these two genes mutated. For 7a10d tumors, these numbers were 40% and 25%, respectively. In contrast, *CIC* and *FUBP1* were relatively frequently mutated in the 1p/19q subgroup (64% and 29%, respectively), but only two *CIC* and no *FUBP1* mutations were observed in the IDHme tumors and none of the 7a10d tumors showed *CIC* and *FUBP1* mutations. Also for *NOTCH1* the IDHme and 7a10d subgroups resemble each other in terms of mutation frequency (7% and 0%, respectively), while about one-fourth of the 1p/19q tumors showed a *NOTCH1* mutation.

### Subgroup 7a10d differs in Verhaak and G-CIMP subtype classification and patient survival from 1p/19q and IDHme

**Fig. 2:**
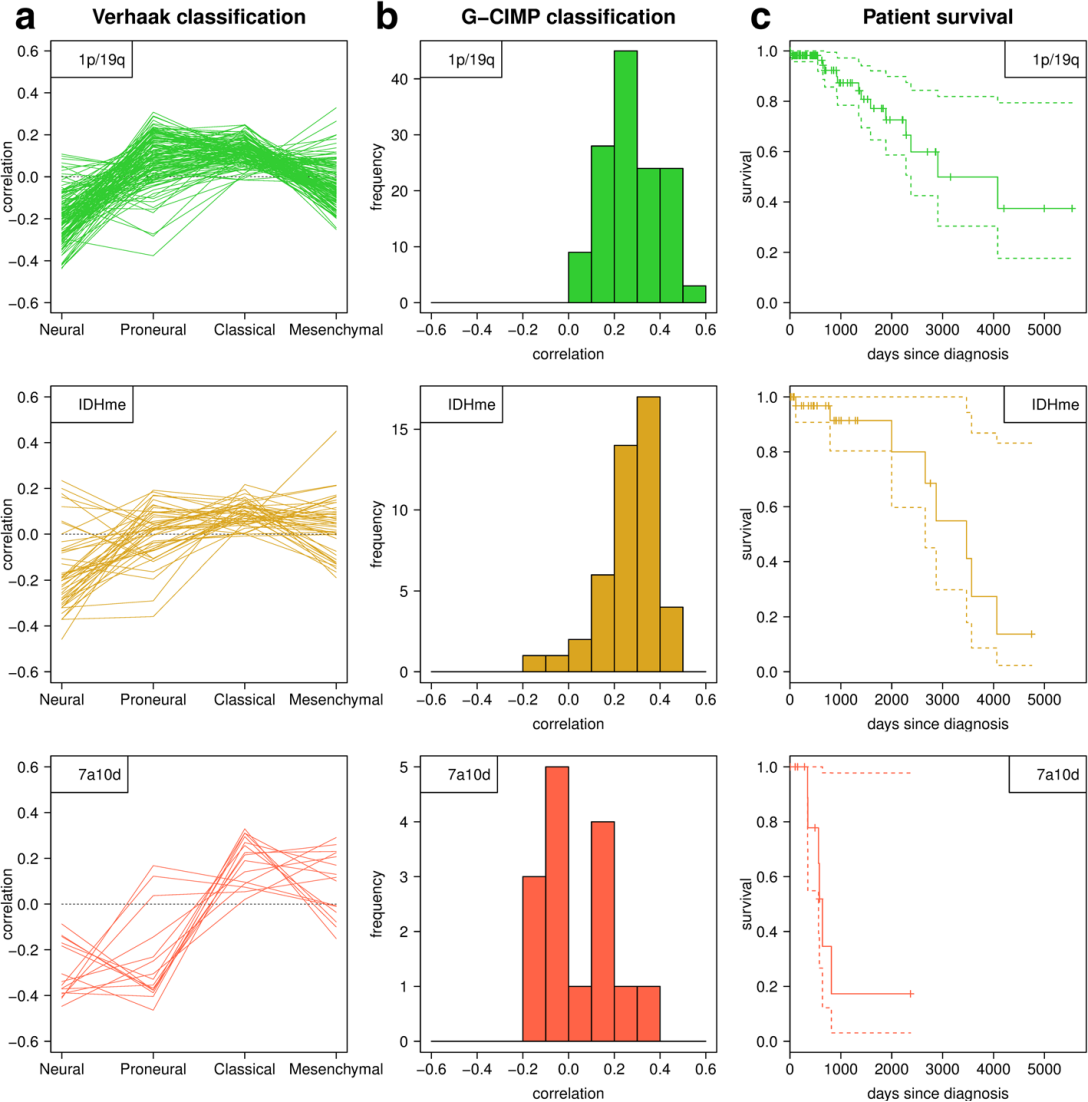
Oligodendroglioma subgroup comparison. **a** Pearson correlation between the expression log-ratios of 756 marker genes in the Verhaak glioma reference set and our tumor subgroups is shown for the four Verhaak classes. **b** Pearson correlation between the expression log-ratios of 42 genes in the G-CIMP reference set and our tumor subgroups. **c** Kaplan-Meier curves (solid lines) and 95%-confidence intervals (dashed lines) of patient survival according to the corresponding clinical data

In order to explore whether tumors of the three oligodendroglioma subgroups differ in their gene expression profiles compared to known molecular glioma subtypes we first considered the Verhaak subtypes [26]. We computed the correlation between the given signature-specific expression levels of the Verhaak subtypes and the corresponding gene expression levels of each individual oligodendroglioma. We observed moderate but still significant correlation values with the Verhaak subtypes for the vast majority of tumors (*P* <0.05 for 130 of 133 1p/19q tumors, for 43 of 45 IDHme tumors, and for all 7a10d tumors considering the Verhaak subtype with the strongest correlation). The 1p/19q and IDHme subgroups showed a similar association pattern (Fig. 2a top and middle). Tumors in both subgroups have highest similarity to the proneural and classical subtypes followed by the mesenchymal subtype, while there is generally a negative correlation with the neural subtype. In contrast, the vast majority of tumors in the 7a10d subgroup had a negative correlation with the proneural subtype (Fig. 2a bottom). This is expected for tumors without an *IDH* mutation [15].

In a similar analysis, we compared the associations of the three oligodendroglioma subgroups with the expression signature of the G-CIMP subtype driven by the mutation of *IDH* [15]. Like for the Verhaak classification, the 1p/19q and IDHme subgroups resembled each other and the tumors in these subgroups had generally positive correlation values to G-CIMP (*P* <0.1 for 73 of 133 1p/19q tumors and 27 of 45 IDHme tumors), as opposed to 7a10d tumors that showed no or very weak positive and negative correlation (*P* <0.1 for 2 of 15 tumors, Fig. 2b).

We also analyzed whether there are differences in patient survival between the three subgroups by using the clinical data available for 125 1p/19q, 34 IDHme, and 15 7a10d tumors. Patients from the 1p/19q and IDHme subgroups showed no differences in survival (Fig. 2c top and middle, log-rank test, *P* = 0.7843). In sharp contrast, patients from 7a10d showed significantly shorter survival than patients from the 1p/19q and IDHme subgroups (Fig. 2c bottom, log-rank tests, *P*=4.9×10^−6^ and *P* = 1.1 × 10 ^−4^, respectively) consistent with previous findings [19].

### All three subgroups are part of known glioma subtypes

Recent studies have defined molecular subtypes for gliomas [19, 20]. We thus analyzed how our three subgroups 1p/19q, IDHme, and 7a10d observed for histologically classified oligodendrogliomas are embedded in these general classification schemes. Diffuse gliomas were grouped into three major subtypes based on the IDH mutation status and the presence of the 1p/19q co-deletion in [19]. Our 1p/19q subgroup corresponds to the 1p/19q subtype in [19]. The IDHme subgroup is included in the subtype that has no 1p/19q co-deletion but an IDH mutation in [19]. The 7a10d subgroup is included in the subtype that has no IDH mutation and no 1p/19q co-deletion, which contains gliomas of which about 50% showed a gain of chromosome 7 and a loss of chromosome 10 [19]. Further, our purely CNV-based derivation of the three subgroups (Fig. 1) shows that tumors with an IDH mutation are more similar to each other than tumors without an IDH mutation. This is in accordance with [19]. Also highly similar gene mutation patterns and survival times are observed for our subgroups and those by [19].

The classification scheme in [19] has been refined in [20] subdividing the IDH mutant group into a G-CIMP-low, G-CIMP-high, and a 1p/19q co-deletion subtype. Our 1p/19q subgroup is included in the 1p/19q co-deletion group in [20]. Further, the vast majority of tumors in our IDHme subgroup belong to the G-CIMP-high group in [20] indicated by the observation of positive correlations with the G-CIMP subtype in our analysis (Fig. 2b middle). Only four IDHme tumors may belong to the G-CIMP-low subtype (correlation with G-CIMP less than 0.1, Fig. 2b middle). This is in good accordance with the molecular classification of histologically classified oligodendrogliomas by [20]. In addition, the non-IDH mutant group was further subdivided in [20] into a classic-like, mesenchymal-like, and two other subtypes. Tumors of our 7a10d subgroup are represented by these subtypes. About half of the 7a10d tumors belong to the classic-like group (Fig. 2a bottom). The majority of the remaining tumors belong to the mesenchymal-like group, but they also show a relatively strong correlation with the classical group (Fig. 2a bottom). This is similar to [20] where also a large proportion of the tumors in the mesenchymal-like group were classified to belong to the classical group of Verhaak [26].

We further tested if the three subgroups were well-embedded in molecular data of closely related histologically classified oligoastrocytomas and astrocytomas of the TCGA lower grade glioma cohort. Therefore, we performed unsupervised clustering of the gene copy number profiles and found that all three subgroups were present among the oligoastrocytomas and that the astrocytomas were split up into the IDHme and 7a10d subgroup. In addition, Verhaak and G-CIMP subtype classifications, patient survival, and gene expression behavior were highly similar between the oligodendroglioma subgroups and corresponding subgroups of oligoastrocytomas and astrocytomas (Additional file 3). This clearly indicates that each of our derived subgroups was adequately covered based on molecular data of histologically classified oligodendrogliomas.

Generally, strong differences in chromosomal mutations, subtype characteristics, and patient survival between the 7a10d subgroup and the other two subgroups 1p/19q and IDHme (Figs. 1 and 2) indicate that 7a10d tumors rather resemble glioblastoma-like tumors [3, 19, 20]. We therefore focused our further analysis on the comparison of tumors from the 1p/19q and IDHme subgroups.

### A signature of differential gene expression discriminates 1p/19q from IDHme

**Fig. 3:**
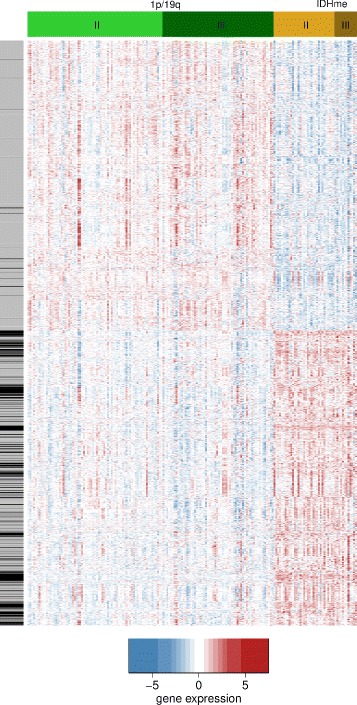
Gene signature distinguishing 1p/19q from IDHme. The heatmap shows the expression values of 5113 differentially expressed (q-value <0.01) signature genes (rows) for 178 histologically classified oligodendrogliomas (columns). Rows are Z-score-scaled and ordered based on a hierarchical clustering of the data (dendrogram not shown). Subgroups are shown in green (1p/19q) and yellow (IDHme) with tumor grades highlighted using light (grade II) and dark (grade III) shadings. Genes that are located on chromosomal arms 1p or 19q are indicated by black lines to the left of the heatmap; other genes are in gray

To compare genome-wide gene expression profiles of the 1p/19q and IDHme subgroups we conducted a differential gene expression analysis contrasting these two subgroups. Using a q-value cut-off of 0.01 we identified 5113 genes to be differentially expressed between 1p/19q and IDHme (Fig. 3, Additional file 4). The expression of half of the signature genes was downregulated in 1p/19q compared to IDHme, while the other half was upregulated. When comparing tumors of grade II and grade III within each subgroup we found no large-scale differences. Only 104 signature genes where differentially expressed between tumor grades II and III for the 1p/19q subgroup (73 grade II vs. 60 grade III tumors, Additional file 5), while there were no significant expression differences of signature genes between tumor grades II and III for the IDHme subgroup (33 grade II vs. 12 grade III tumors).

### The signature is enriched for signaling and metabolic pathway genes and transcription factors

**Fig. 4:**
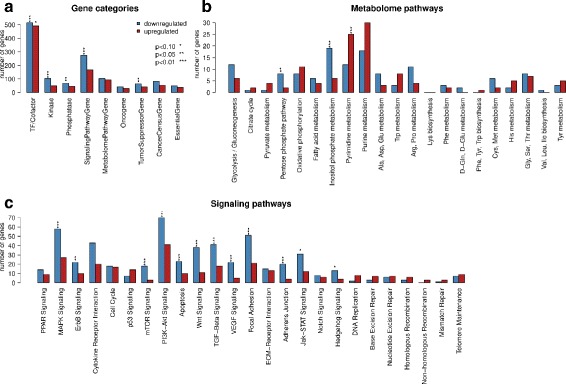
Functional analysis of signature genes. Enrichment of gene categories (**a**), metabolic pathways (**b**), and signaling pathways (**c**) with signature genes. Bars are shown separately for genes down- and upregulated in 1p/19q compared to IDHme using blue and red color, respectively. Significance of enrichment was calculated using Fisher’s exact test and is highlighted by asterisks symbols

Looking at the annotations of the 5113 signature genes we found that the categories transcription factor/cofactor, kinase, phosphatase, signaling pathway gene, and tumor suppressor gene were significantly enriched for downregulated genes in tumors of the 1p/19q subgroup compared to that of the IDHme subgroup (*P* <0.05, Fig. 4). For signature genes upregulated in 1p/19q compared to IDHme only the transcription factor/cofactor category was found to be significantly enriched (*P* <0.1). Among the affected signaling pathways several pathways known to be involved in cancer were significantly enriched with genes downregulated in 1p/19q tumors compared to IDHme (Fig. 4c). These were the MAPK signaling, ErbB signaling, mTOR signaling, PI3k-Akt signaling, Apoptosis, Wnt signaling, TGF-Beta signaling, VEGF signaling, Focal adhesion, Adherence junction, Jak-STAT signaling, and Hedgehog signaling pathway, which are known to affect proliferation, differentiation, migration, adhesion, cell growth and survival, cell cycle arrest and progression, and metabolism (see Table S4 in [33]). For genes upregulated in 1p/19q no enrichment of signaling pathways was observed.

Regarding metabolic pathways (Fig. 4b), the pentose phosphate pathway (generating NADPH, pentoses, and Ribose 5-phosphate, a precursor for nucleotide synthesis) and inositol phosphate pathway (generating inositol phosphates that play a role in various cellular processes including cell growth and differentiation, cell migration and apoptosis) were significantly enriched with genes downregulated in 1p/19q (*P* <0.05 and *P* <0.01, respectively). The pyrimidine pathway (generating cytosine, thymine, and uridine nucleotides) was enriched with genes showing an increased expression in 1p/19q tumors (*P* <0.01).

Moreover, there were in total 1006 transcription factors/cofactors present in the signature (Additional file 6), forming the basis for the subsequent reconstruction of a gene regulatory network that is associated with the observed expression differences between the 1p/19q and IDHme subgroups.

### A gene regulatory network is associated with expression differences between 1p/19q and IDHme

**Fig. 5:**
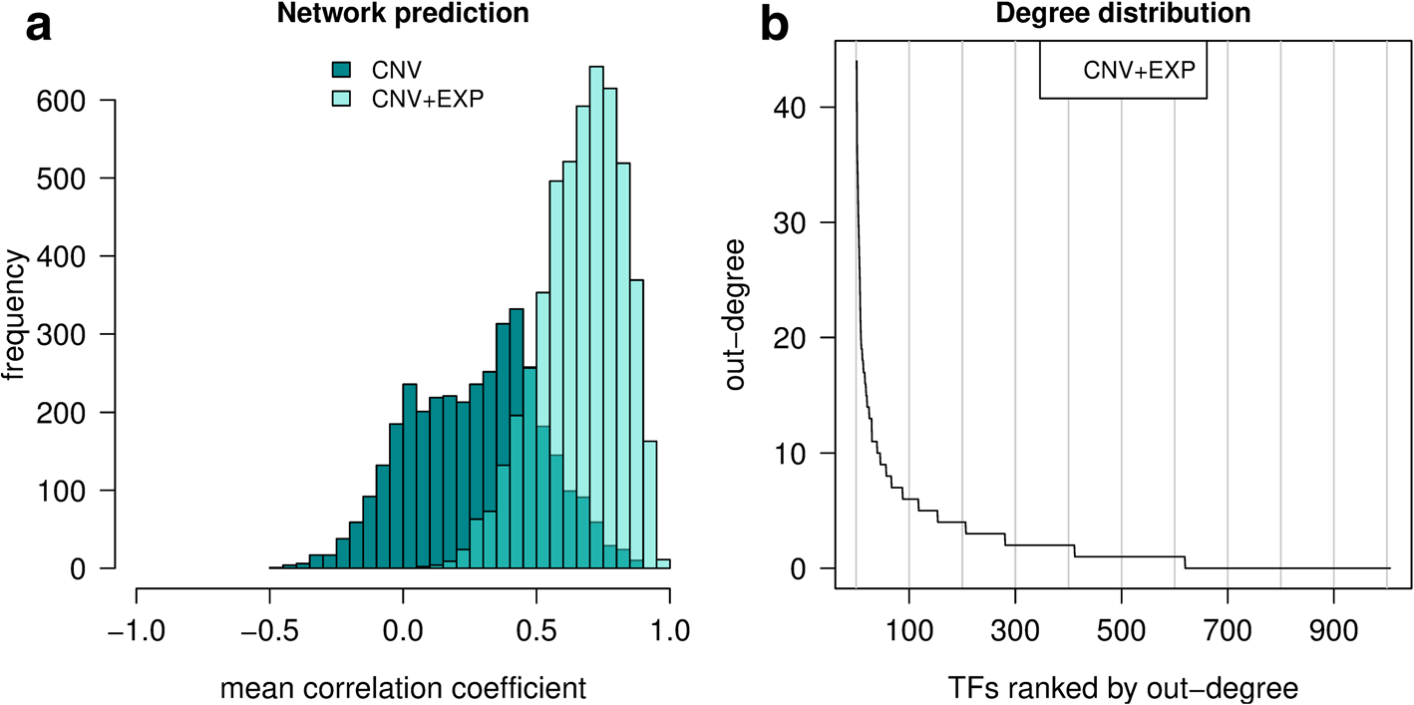
Regulatory network predictions and hub transcription factors. **a** Pearson correlations between predicted and measured expression of the 5113 signature genes on the test data are shown for the network utilizing only gene copy number data (CNV: dark-turquoise) and the network utilizing both gene copy number and gene expression data (CNV + EXP: light-turquoise) as predictors. Correlation values are averaged over 100 cross-validation iterations (see Methods for details). **b** Ranking of transcription factors by out-degree for the CNV + EXP network. Only very few transcription factors have high out-degree values (hubs), whereas the large majority shows few connections to other signature genes

We sought to construct a gene regulatory network which can predict the expression of the 5113 signature genes distinguishing 1p/19q from IDHme. In this analysis, 100 cross-validated networks were computed and used to calculate an average predicted expression value for each signature gene (see “Methods” for details). We applied the approach to two different predictor sets. First, we started to learn a network for which only the copy number of a gene was used to predict its expression. For 1442 signature genes (28.2%) no prediction of gene expression based on the underlying gene copy number was obtained. For the vast majority of the remaining signature genes the average predicted expression value correlated positively with the measured expression for the test data (Fig. 5a), and the median correlation coefficient over all signature genes was 0.292 (*P* <0.05 for 53.7% of the genes).

In the second analysis, we learned a regulatory network by utilizing both the gene-specific copy numbers and the expression values of transcription factors that were part of the signature as predictors. This network yielded significantly better predictions than the CNV-only network (Fig. 5a, Mann-Whitney U test, *P*≈0). Predictions were obtained for all signature genes, and the median correlation coefficient was 0.676 on the test data (*P* <0.05 for 95.8% of the genes). We chose this second network (Additional file 7) for further analysis because of its superior prediction accuracy and the possibility to identify potential regulators of other signature genes.

**Fig. 6:**
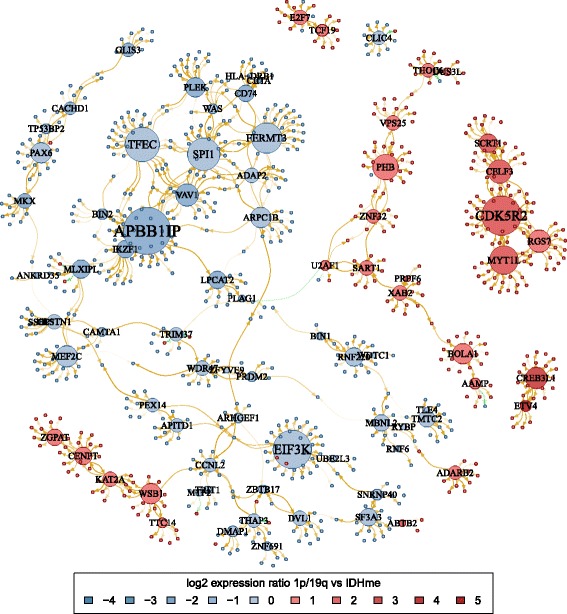
Gene regulatory network distinguishing 1p/19q from IDHme Sub-network showing transcription factors with an out-degree of at least 10 and the signature genes connected to them. Genes (nodes) are colored according to gene expression log-ratios between the average expression in 1p/19q and IDHme. Nodes with labels represent transcription factors and the size of a node is proportional to its out-degree. Activating and repressing links are shown in yellow and green color, respectively, and transparency of a link is inversely proportional to the number of times the link is present across the 100 cross-validation iterations

Hubs in the network, e.g. nodes with high degree that have many connections to other nodes, may help to identify potential key regulators involved in the manifestation of differences between the 1p/19q and IDHme subgroups. We thus looked at the out-degree of nodes representing transcription factors and found that few of them (49 of 1006, 4.9%) had an out-degree of at least 10, while the vast majority were connected to few signature genes (Fig. 5b). A sub-network containing only these hub transcription factors and the signature genes connected to them by ingoing or outgoing links is shown in Fig. 6. The vast majority of network connections represent activating links. Moreover, this sub-network can be further partitioned into potential gene regulatory modules that (i) show many internal connections, (ii) have few or no external links to other gene clusters, and (iii) comprise signature genes with comparable patterns of expression differences between 1p/19q and IDHme (see node coloring in Fig. 6).

### Regulatory hubs and gene network modules affect cancer-relevant functions

One of the gene modules in our regulatory network (Fig. 6) contains *APBB1IP*, the gene with the highest out-degree in the network, as well as other hub transcription factors including *VAV1*, *ARPC1B*, *SPI1*, *TFEC*, *FERMT3*, and *IKZF1*, among others. The expression of genes in this cluster is downregulated in 1p/19q compared to IDHme. According to GeneCards [34] and UniProtKB/Swiss-Prot [35] annotations, *APBB1IP* functions in signal transduction from Ras activation to actin cytoskeletal remodeling [36, 37], *VAV1* is a guanine nucleotide exchange factor for Rho family GTPases also known to be involved in the regulation of cytoskeletal rearrangements and a known proto-oncogene [38], *ARPC1B* regulates actin polymerization and mediates the formation of branched actin networks [39], *SPI1* is a proto-oncogene potentially involved in the regulation of pre-mRNA splicing [40], *TFEC* has been associated with breast cancer and is part of the cancer-related C-MYB transcription factor network [41], *FERMT3* has been associated with cell adhesion deficiencies [42], and *IKZF1* is known to be involved in different types of cancer [43].

A second gene module includes the hub transcription factors *CDK5R2*, *MYT1L*, *CELF3*, *RGS7*, and *SCRT1* (Fig. 6). In contrast to the first gene cluster described above, the expression of genes in this second cluster is upregulated in 1p/19q compared to IDHme. *CDK5R2* is a regulator of the cell division protein Cyclin-dependent kinase 5 and has been associated with neuronal migration and development [44], *MYT1L* is a pan-neural transcription factor involved in neuronal differentiation and is thought to play a role in the development of neurons and oligodendroglia [35], *CELF3* is involved in the regulation of pre-mRNA alternative splicing [45], *RGS7* is associated with benign neoplasms in different organs and regulates G-protein-coupled receptor signaling [46], and *SCRT1* is a Zinc finger DNA-binding protein critical for neuronal differentiation [47].

There are other individual hub transcription factors in the network with potentially relevant functions in cancer development. One of them is *PHB* (upregulated in 1p/19q compared to IDHme) that codes for prohibitin, which inhibits DNA synthesis, has been associated with breast cancer, and plays a role in regulating proliferation [48, 49]. *CREB3L1* (upregulated in 1p/19q) is thought to be involved in the protection of astrocytes from ER stress-induced cell death [50]. *CENPT* (upregulated in 1p/19q) encodes one of the inner kinetochore proteins and is required for normal chromosome organization and progress through mitosis [51]. *MEF2C* (dowregulated in 1p/19q) is crucial for normal neuronal development and has been suggested to be involved in neurogenesis and in the development of cortical architecture [52, 53]. *EIF3K* (downregulated in 1p/19q) is a component of the eukaryotic translation initiation complex regulating protein synthesis [54]. *CCNL2* (downregulated in 1p/19q) regulates a critical factor involved in cell apoptosis [55]. Further, *ETV4* involved in developmental processes and oncogenesis [34] was upregulated in 1p/19q compared to IDHme.

### Comparison of 1p/19q and IDHme to closely related oligodendrogliomas and astrocytomas

Recently, bulk and single cell transcriptomes of *IDH*-mutant oligodendrogliomas and astrocytomas have been compared [22]. This study suggested shared glial lineages and developmental hierarchies where most differences resulted from characteristic mutations and microenvironmental compositions. In more detail, they observed that differences in bulk gene expression profiles between oligodendrogliomas and astrocytomas can be primarily explained by the impact of characteristic tumor class-specific mutations (oligodendrogliomas: 1p/19q co-deletion, *CIC* mutations; astrocytomas: *TP53* mutations) and differences in the composition of the tumor microenvironment, but not by distinct expression programs of glial lineages of malignant cells. They compared oligodendrogliomas defined based on their histology and the presence of the 1p/19q co-deletion to astrocytomas defined based on their histology and the presence of mutations in *TP53* or *ATRX*. This is similar to our analysis. Our 1p/19q subgroup has the same histological and genetic features as their oligodendrogliomas. Our IDHme subgroup is closely related to their astrocytomas, except for differences in histology. In accordance with [22], we observed downregulations of genes on 1p and 19q (Fig. 3) and upregulations of genes of the p53 signaling pathway (Fig. 4c) in 1p/19q compared to IDHme. We found similar evidences that genes involved in cytoskeleton remodeling (e.g. *APBB1IP*, *VAV1*, *ARPC1B*, Fig. 6) were downregulated in 1p/19q compared to IDHme, which might indicate potentially existing morphological differences. Further, we found significant expression differences between 1p/19q and IDHme analyzing oligodendrocyte-like and astrocyte-like expression programs from [22] (Additional file 8: Figure S1A-B, t-test, *P* = 4.8 × 10 ^−11^). The 1p/19q subgroup showed higher expression of genes of the oligodendrocyte-like program than the IDHme subgroup, whereas IDHme showed higher expression of genes of the astrocyte-like program. Similarly, both groups also differed in their expression of microglia/macrophage marker genes (Additional file 8: Figure S1C, t-test, *P* <0.03). Interestingly, we found a weak trend that the 1p/19q and IDHme subgroups differ in the expression of the stemness program from [22]. Still, the majority of genes of the stemness program showed similar expression levels in both groups, but there were several genes with stronger expression differences (Additional file 8: Figure S1D). This included genes involved in cytoskeleton remodeling (absolute average log-ratio for 1p/19q compared to IDHme >1; *DCX*,*TMSB15A*: upregulated in 1p/19q; *FNBP1L*: downregulated in 1p/19q) and *MYT1L*, a known key factor of neural differentiation, upregulated in 1p/19q compared to IDHme.

### 1p/19q and IDHme tumors differ in stemness programs

**Fig. 7:**
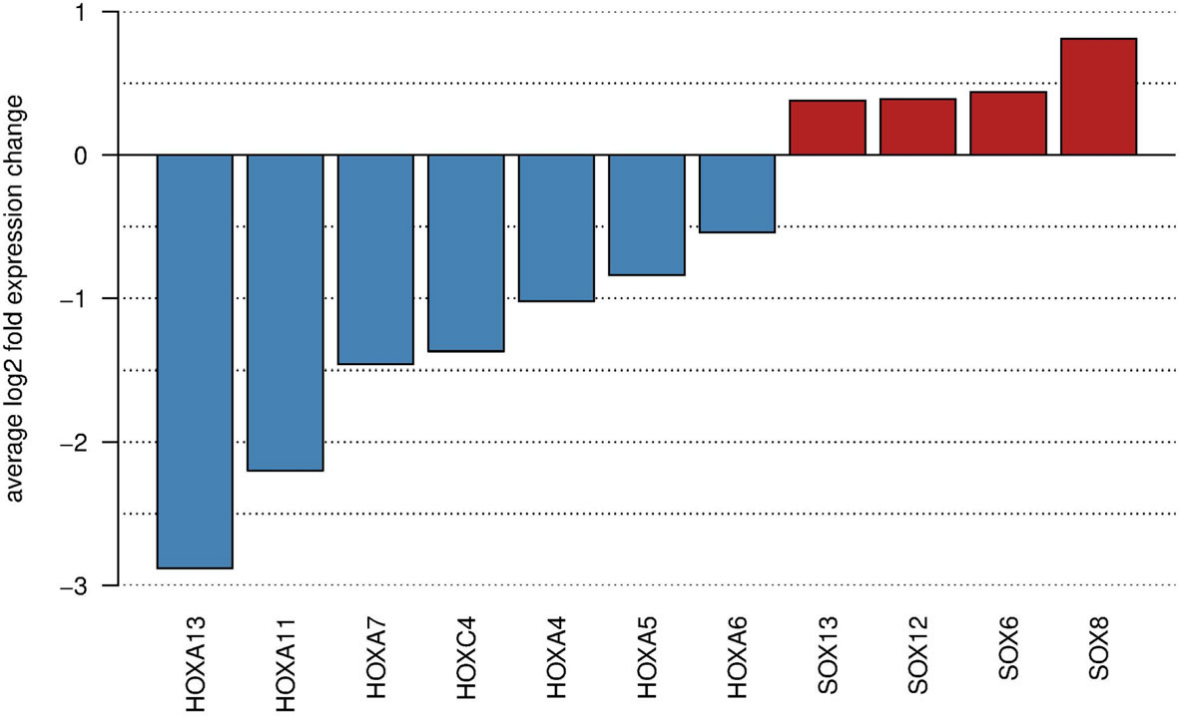
HOX and SOX signature distinguishing 1p/19q from IDHme. Average log-fold expression differences of *HOX* and *SOX* genes between the 1p/19q and the IDHme subgroup. Genes downregulated in 1p/19q compared to IDHme are shown in blue and upregulated genes are shown in red. Gene expression differences between tumors of both groups were highly significant with q-values clearly less than 0.01 (Additional file 6)

Glioma stemness programs have been characterized over the last years suggesting important regulatory roles for different members of the *HOX* [20, 56] and *SOX* [20–22, 57] gene families. Roles of *SOX* genes in development and pathology have been reviewed in [58]. We thus analyzed our regulatory network (Additional file 7) for characteristic expression differences of both gene families between 1p/19q and IDHme. Our network includes seven *HOX* genes (*HOXA4*, *HOXA5*, *HOXA6*, *HOXA7*, *HOXA11*, *HOXA13*, *HOXC4*) and four *SOX* genes (*SOX6*, *SOX8*, *SOX12*, *SOX13*). Interestingly, all *HOX* genes were downregulated in 1p/19q compared to IDHme, whereas all *SOX* genes were upregulated in 1p/19q compared to IDHme (Fig. 7). This indicates the activity of different stemness programs between 1p/19q (potentially *SOX*-driven) and IDHme (potentially *HOX*-driven) tumors.

Moreover, this is also supported by already known cancer-relevant functions of different genes. *HOXA4* overexpression suppressed cell motility and spreading in ovarian cancer [59]. *HOXA5* downregulation increased stemness, cell plasticity and aggressiveness of breast cancer [60], and upregulation induced stemness loss in colon cancer [61]. *HOXA7* overexpression enhanced proliferation, migration, invasion and metastasis of liver cancer [62]. *HOXA11* was reported to represent a potential tumor suppressor in different cancers [63, 64]. *HOXC4* overexpression of was observed in lymph node metastases of prostate cancer [65]. Interestingly, different *SOX* genes have already been reported to be involved in oligodendrocyte development. Alterations of corresponding gene expression patterns can therefore be important for tumor development. *SOX6* regulates different stages of oligodendrocyte development by repressing cell specification and terminal differentiation and by influencing cell migration patterns [66]. *SOX8* is expressed in immature glia of the developing cerebellum and in cerebellar tumors [67] and has important functions in oligodendrocyte development and differentiation [68, 69]. *SOX13* regulates the differentiation of specific neurons [70].

## Discussion

First, we analyzed gene copy number data of histologically classified oligodendrogliomas from TCGA and revealed three molecular subgroups by hierarchical clustering of gene copy number data alone (Fig. 1). We used additional information about the presence of a 1p/19q co-deletion [8] and an *IDH* mutation [11] to further characterize these subgroups. In accordance with previous findings for histologically classified oligodendrogliomas [10, 71] and gliomas in general [19], we observed a large 1p/19q subgroup characterized by concurrent 1p/19q co-deletion and *IDH* mutation, an intermediate IDHme subgroup of tumors that mainly show an *IDH* mutation but no commonly overrepresented gene copy number alterations, and a small 7a10d subgroup showing a concurrent duplication of chromosome 7 and a deletion of chromosome 10 where most tumors lacked *IDH* mutations. In addition, considering Verhaak [26] and G-CIMP [15] classes, the 1p/19q and the IDHme subgroup resembled each other, whereas the 7a10d subgroup strongly deviated from these two subgroups also in terms of significantly lower overall patient survival (Fig. 2). This, in combination with the molecular characteristics of the 7a10d subgroup, suggests that these tumors might rather represent glioblastoma-like tumors [3]. This is also supported by a refined molecular classification of gliomas in [20]. Thus, tumors of our small 7a10d subgroup may have been falsely classified as oligodendrogliomas based on histology alone, which is not unlikely considering difficulties of pure histological classifications [6]. We therefore decided to focus our further analyses on the comparison of the 1p/19q and the IDHme subgroups.

Second, we performed an in-depth analysis of the 1p/19q and IDHme subgroups deriving a characteristic gene expression signature that distinguished tumors of both groups (Fig. 3). Interestingly, many of these signature genes were part of signaling pathways involved in the regulation of cell proliferation, differentiation, migration, and cell-cell contacts (Fig. 4). Several of these pathways have already been reported to be involved in glioma development (e.g. PI3K-AKT, MAPK, VEGF signaling) [27, 33, 72, 73]. The strong downregulation of these pathways in the 1p/19q subgroup compared to the IDHme subgroup might be associated with a better sensitivity to treatment and prognosis of (1p/19q) oligodendrogliomas compared to other low-grade gliomas [74, 75].

Third, to better understand differences between the 1p/19q and the IDHme subgroup, we reconstructed a gene regulatory network capable to explain gene expression differences between both subgroups (Figs. 5 and 6). Interestingly, we revealed that several potential hub transcription factors involved in remodeling of the cytoskeleton (e.g. *APBB1IP*, *VAV1*, *ARPC1B*), apoptosis (*CCNL2*, *CREB3L1*), and neural development (e.g. *MYTIL*, *SCRT1*, *MEF2C*) were differentially expressed between both subgroups. Since all or the vast majority of tumors of these two subgroups show *IDH* mutations, the globally observed expression differences are likely to be strongly influenced by the 1p/19q co-deletion. Moreover, we observed characteristic expression differences between *HOX* and *SOX* transcription factors (Fig. 7). All *HOX* genes included in our network were downregulated and all *SOX* genes were upregulated in 1p/19q compared to IDHme. This indicates that the 1p/19q subgroup and the IDHme subgroup express different stemness programs. Recent findings of specific *HOX* and *SOX* gene expression patterns for different types of gliomas indicate an important role of both gene families in brain tumors [20–22]. This is also supported by the recent finding that *SOX2* repression is an early driver of gliomagenesis that blocks the differentiation of neural stem cells in an *in-vitro* model of low-grade astrocytomas [76]. Further experimental studies are required to analyze our revealed stemness signatures.

Finally, it is important to discuss the revealed molecular subtypes in the light of the new WHO 2016 brain tumor classification scheme [18]. All oligodendrogliomas that we analyzed have been classified by the TCGA according to the WHO 2007 brain tumor classification scheme [4], which was state-of-the-art when the tumors were obtained. This older classification is purely based on histology, whereas the new WHO 2016 classification additionally considers the 1p/19q-co-deletion and the *IDH* mutation status. There would be differences in the grouping of tumors, but a reclassification of the analyzed tumors is not straightforward and would require expert knowledge of neuropathologists that have to consider histological and molecular data. Therefore, we cannot realize this reclassification for the considered TCGA data set, but we can interpret our subgroups with respect to the new WHO 2016 classification. Considering our 7a10d subgroup, information about the gain of chromosome 7 and the deletion of chromosome 10 are not considered at all in the new WHO 2016 classification system [18]. Thus, tumors of these subgroup would still not be classified as glioblastomas if no clear signs of high malignancy (necrosis, pathological vascular proliferation) are observed in histology. It is likely that such signs were not present in nearly half of the 7a10d tumors (6 of 15), otherwise these tumors would have been assigned the WHO grade IV instead of grade II according to the WHO 2007 brain tumor classification system. Therefore, these tumors of our 7a10d subgroup might rather be classified as astrocytoma *IDH*-wildtype or *IDH*-mutant (if histological and molecular data are conclusive) or even as oligodendroglioma, NOS (if histological and molecular data are inconclusive) according to the WHO 2016 brain tumor classification system. This may change in future [77]. Such low-grade gliomas without any signs of high malignancy and without *IDH* mutation still represent an area of ongoing research [78]. Further, like for the WHO 2007 brain tumor classification, all tumors of our 1p/19q subgroup would also be classified as oligodendrogliomas (*IDH*-mutant and 1p/19q-codeleted) according to the WHO 2016 brain tumor classification system. This is also supported by the characteristic overexpression of *SOX* genes. In contrast, tumors of our IDHme subgroup would now be classified as astrocytoma *IDH*-mutant or *IDH*-wildtype also when oligodendroglia-like features are present in histology. This is further supported by the presence of characteristic *ATRX* (30 of 45 tumors) or *TP53* (35 of 45 tumors) mutations in *IDH*-mutated tumors [18]. It is important to note that the new WHO 2016 brain tumor classification system does not change the results of our study. The observed molecular differences between subgroups exist independent of the underlying classification system. Still, one should always be aware of the underlying classification system. In the light of the new WHO 2016 brain tumor classification system, we performed an in-depth comparison of oligodendrogliomas (*IDH*-mutant and 1p/19q co-deleted) represented by our 1p/19q subgroup to astrocytomas (vast majority *IDH*-mutant) represented by our IDHme subgroup. This is supported by our finding that the 1p/19q subgroup expressed an oligodendrocyte-like program and that the IDHme subgroup expressed an astrocyte-like program [22].

## Conclusions

Our study confirms prior findings about the molecular subtyping of histologically classified oligodendrogliomas and further provides novel insights into gene expression differences between subtypes. It is important to note that we were able to derive these subtypes purely based on gene copy number data alone. Additional information about the presence of a 1p/19q co-deletion and an *IDH* mutation were only considered subsequently to further characterize these subgroups. The in-depth comparison of the 1p/19q and IDHme subgroups provides novel insights into differences at the level of single genes, pathways, and regulatory networks that have not been reported so far. We identified a characteristic gene expression signature that distinguishes both subgroups including several known signaling pathways that impact on cell proliferation, migration, and angiogenesis. We derived a gene regulatory network that can explain expression differences between both subgroups. Our network-based analysis enabled us to predict novel putative major regulators that contribute to the manifestation of differences between both subgroups. Several of these major regulators are known to be involved in the regulation of cytoskeleton remodeling, apoptosis, and neural development. Moreover, we also revealed a characteristic *HOX* and *SOX* gene expression signature that distinguishes both subgroups suggesting the activity of different glioma stemness programs.

Further, the analyzed oligodendroglioma data set represents an important resource for future research, but researchers have to be aware that these tumors were classified by TCGA according to the WHO 2007 brain tumor classification system. We hope that the discussion of our findings in the context of the new WHO 2016 classification will raise awareness for the fact that brain tumor classification systems can vary considerably. This is important for the interpretation of the results of our retrospective study and for future studies based on the considered TCGA data set.

In summary, our in-depth study focused on the analysis of molecular data of histologically classified oligodendrogliomas. Especially with respect to an oligodendroglial phenotype, characteristic expression differences associated with histological classification may also exist for other types of gliomas. Future studies with already existing molecular data of histologically classified oligodendrogliomas, oligoastrocytomas, and astrocytomas could search for such patterns and evaluate their value for molecular tumor classification.

## Additional files

Additional file 1 Oligodendroglioma TCGA identifiers and survival information. (XLSX 14.1 kb)

Additional file 2 Gene copy number and gene expression data of oligodendrogliomas and gene expression data of normal brain references. (XLSX 35942 kb)

Additional file 3 Comparison of revealed subtypes to subtypes revealed for histologically classified oligoastrocytomas and astrocytomas. (PDF 2365 kb)

Additional file 4 Normalized expression values of 5113 signature genes. (TXT 6266 kb)

Additional file 5 104 signature genes differentially expressed between tumor grades II and III. (XLSX 13.7 kb)

Additional file 6 1006 transcription factors of the gene signature. (XLSX 92.4 kb)

Additional file 7 Consensus gene regulatory network. Rows of the matrix represent 5113 response variables (signature genes); columns represent 1007 predictors (transcription factors + gene-specific copy number). A cell’s value shows how many times the link from the respective predictor gene to the respective response gene was present across the 100 cross-validation iterations. Negative values indicate repressing links and positive values indicate activating links. (TXT 10209 kb)

Additional file 8 **Figure S1.** Comparison to Venteicher et al. (PDF 49.5 kb)

## Abbreviations

CNV: Gene copy number variation
G-CIMP: Glioma-CpG island methylator phenotype
IDH: Isocitrate dehydrogenase
OD: Oligodendroglioma
TCGA: The Cancer Genome Atlas
TF: Transcription factor
WHO: World Health Organization
1p/19q subgroup: Tumors with 1p/19q co-deletion and IDH mutation
IDHme subgroup: Tumors that predominantly show IDH mutations but no 1p/19q co-deletion
7a10d subgroup: Tumors that show an amplification of chromosome 10 and a deletion of chromosome 7 but mostly no IDH mutation
