# Supplementary material for: Comparative analysis of histologically classified oligodendrogliomas reveals characteristic molecular differences between subgroups
Source: BMC Cancer. 2018 Apr 10;18:399. doi: 10.1186/s12885-018-4251-7 (PMC5892046; doi:10.1186/s12885-018-4251-7)
Supplement: Supplementary file 3 — Comparison of revealed subtypes to subtypes revealed for histologically classified oligoastrocytomas and astrocytomas. (PDF 2365 kb) [file 12885_2018_4251_MOESM3_ESM.pdf]

Comparison of revealed oligodendroglioma and oligoastrocytoma subgroups

We analyzed the 131 histologically classified oligoastrocytomas from the TCGA LGG cohort for the presence of our molecular subgroups observed for oligodendrogliomas. Again, we used unsupervised clustering based on gene copy number profiles revealed that oligoastrocytomas can also be split up into three subgroups that are very similar to those observed for the oligodendrogliomas (Figure 1). The oligoastrocytoma 1p/19q subgroup comprised 34 tumors while the IDHme and 7a10d subgroups included 84 and 13 tumors, respectively. This grouping of oligoastrocytoma samples was also recovered when we applied our regulatory network to the oligoastrocytoma gene copy number and gene expression data. For the oligoastroctyoma 1p/19q, IDHme, and 7a10d subgroups the expression of the signature genes can be predicted with an accuracy that is comparable to those obtained for the corresponding oligodendroglioma 1p/19q, IDHme, and 7a10d subgroups (Figure 2A). Likewise, the subgroup-based patterns in Verhaak classification (Figure 2B) and G-CIMP classification (Figure 2C) were comparable between oligodendrogliomas and oligoastrocytomas. This was also observed for the survival of patients of the IDHme and 7a10d subgroups, but not for the 1p/19q subgroup for which none of the corresponding oligoastrocytoma patients was documented as deceased (Figure 2D).

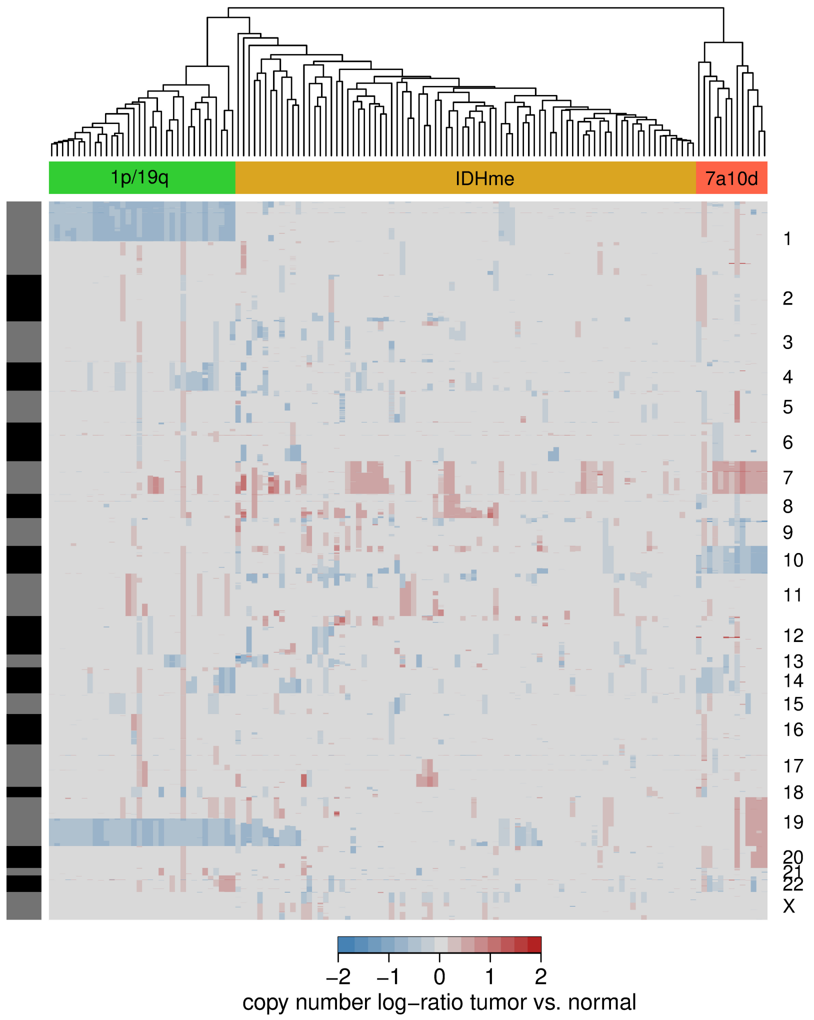

Figure 1. Clustering of 132 histologically classified oligoastrocytomas based on genome-wide gene copy number log-ratio profiles. Tumors width an IDH1/2 mutation per oligoastrocytoma subgroup: 100% (34 of 34, 1p/19q), 95.2% (80 of 84, IDHme), 7.7% (1 of 13, 7a10d). For further details see legend of Fig. 1 of the main manuscript.

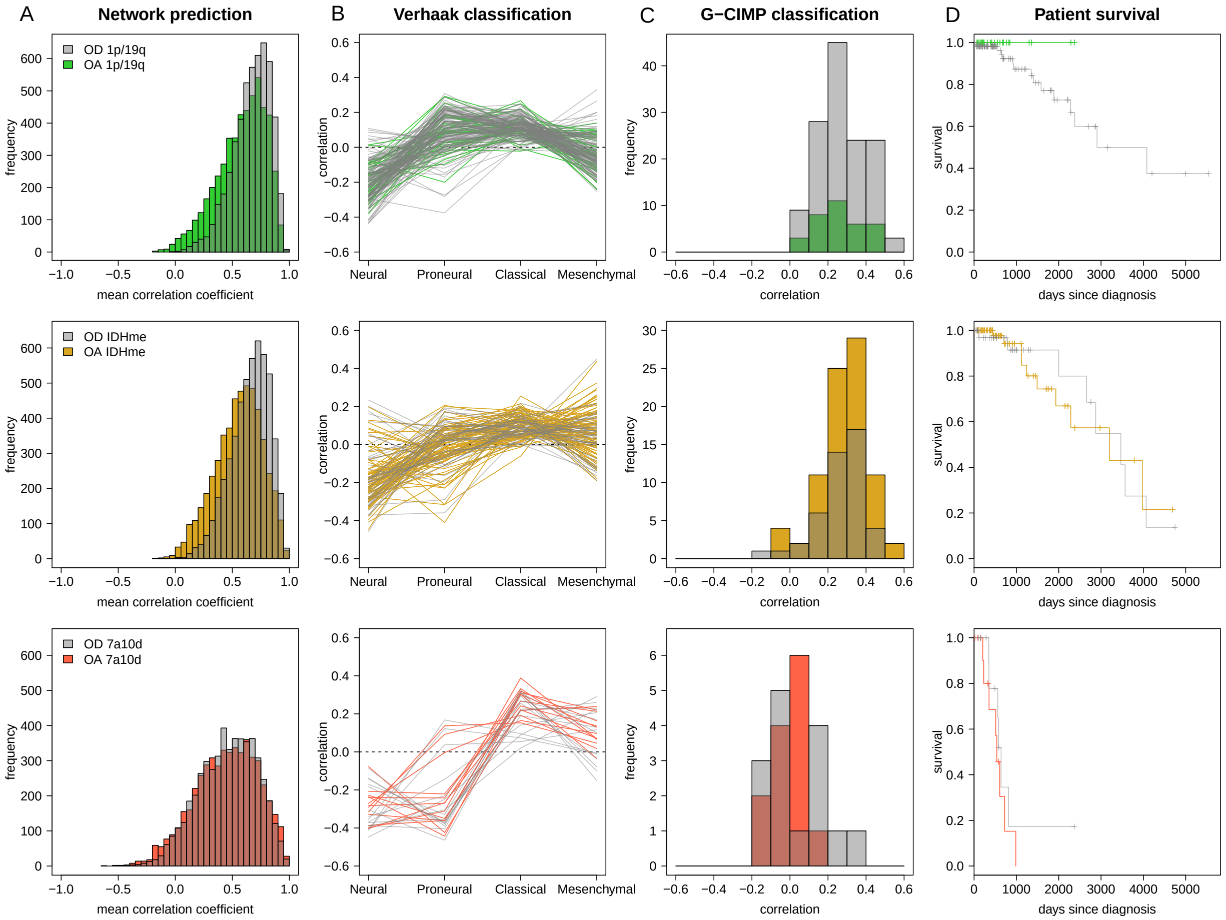

Figure 2. Comparison of revealed oligodendroglioma and oligoastrocytoma subgroups. A) Pearson correlation of network-predicted and experimentally measured expression levels of the 5113 signature genes for oligodendroglioma (gray) and oligoastrocytoma (green: 1p/19q, yellow: IDHme, red: 7a10d) subgroups. Verhaak classification (B), G-CIMP classification (C), and patient survival (D) are very similar for each pair of subgroups. P-values of log-rank tests comparing the survival curves in D: 0.175 (top), 0.924 (middle), 0.37 (bottom). See also legend of Fig. 2 of the main manuscript for further details. OD: oligodendroglioma, OA: oligoastrocytoma.

Comparison of revealed oligodendroglioma and astrocytoma subgroups

We further extended our analysis to the 197 histologically classified astrocytomas from the TCGA LGG cohort. We observed that they can be split up into two subgroups that are very similar to the IDHme and 7a10d subgroups observed for oligodendrogliomas and oligoastrocytomas (Figure 3). These two astrocytoma subgroups strongly resembled the Verhaak and G-CIMP classification and overall patient survival as observed for the oligodendroglioma subgroups (Figure 4).

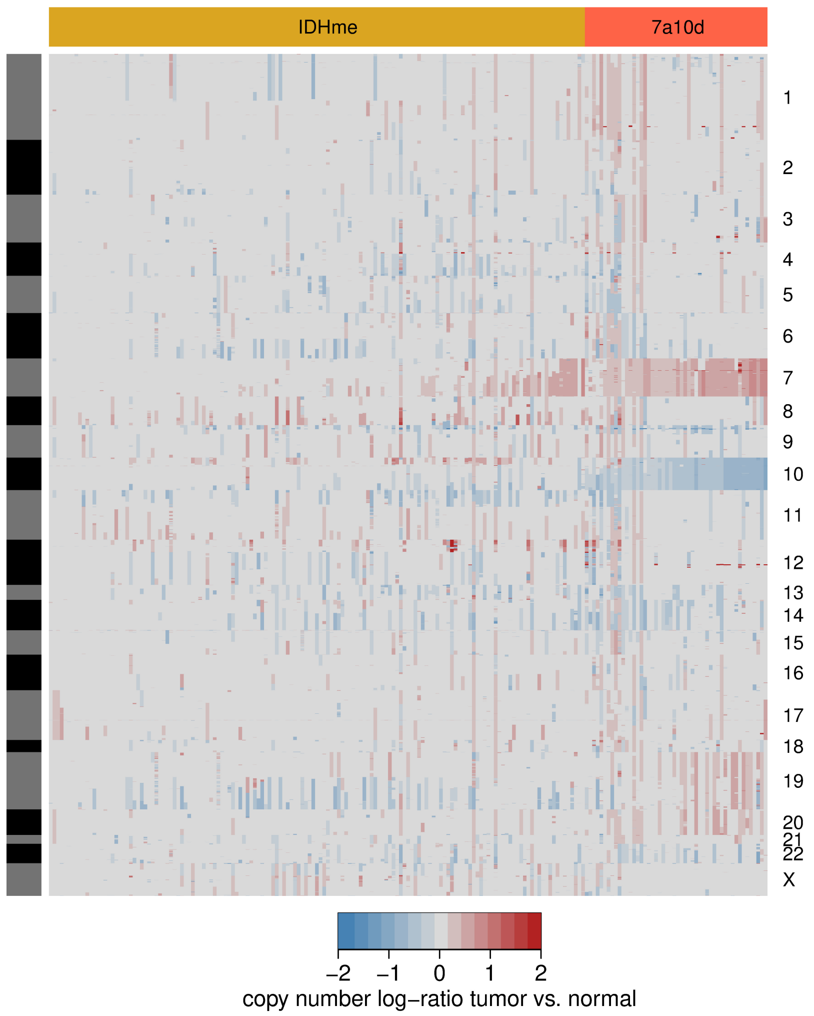

Figure 3. Clustering of 197 histologically classified astrocytomas based on copy number log-ratio profiles of genes on chromosomes 7 and 10. Samples were assigned to the 7a10d subgroup if the mean copy number log-ratio of genes on chromosome 7 was larger than 0.1 and the mean copy number log-ratio of genes on chromosome 10 was smaller than -0.1; other samples were assigned to the IDHme subgroup. Columns are ordered by subgroup. Tumors width an IDH1/2 mutation per astrocytoma subgroup: 89.1% (131 of 147, IDHme), 16.0% (8 of 50, 7a10d). For further details see legend of Fig. 1 of the main manuscript.

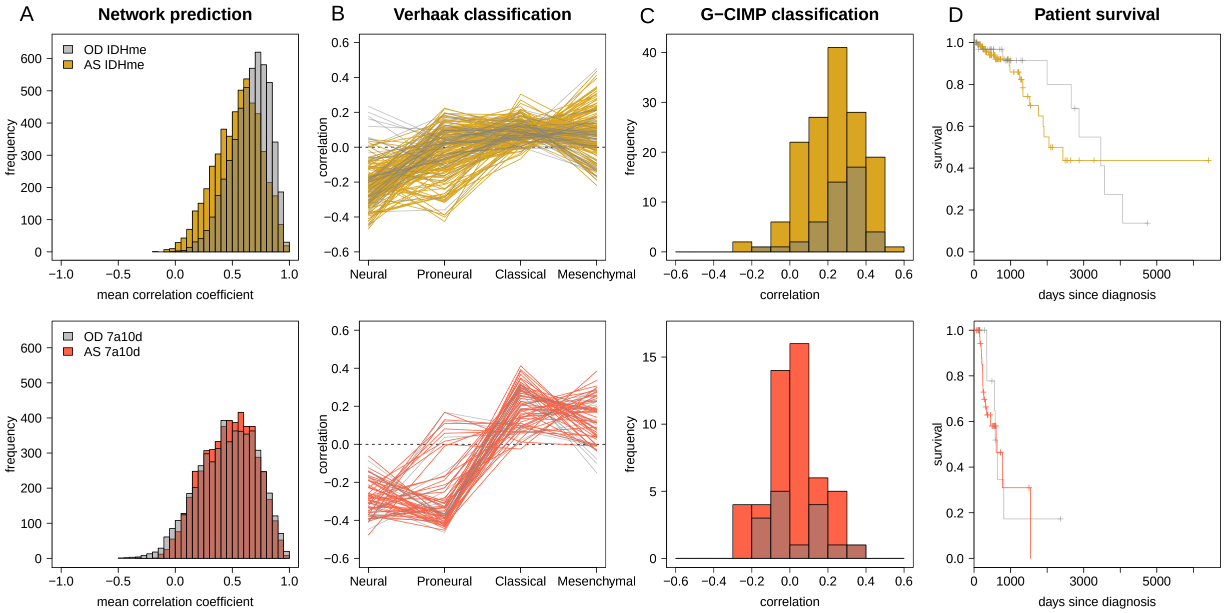

Figure 4. Comparison of revealed oligodendroglioma and astrocytoma subgroups. A) Pearson correlation of network-predicted and experimentally measured expression levels of the 5113 signature genes for oligodendroglioma (gray) and astrocytoma (yellow: IDHme, red: 7a10d) subgroups. Verhaak classification (B), G-CIMP classification (C), and patient survival (D) are very similar for each pair of subgroups. P-values of log-rank tests comparing the survival curves in D: 0.402 (top), 0.569 (bottom). See also legend of Fig. 2 of the main manuscript for further details. OD: oligodendroglioma, AS: astrocytoma.
