# Supplementary material for: Comparative analysis of histologically classified oligodendrogliomas reveals characteristic molecular differences between subgroups
Source: BMC Cancer. 2018 Apr 10;18:399. doi: 10.1186/s12885-018-4251-7 (PMC5892046; doi:10.1186/s12885-018-4251-7)
Supplement: Supplementary file 8 — Figure S1. Comparison to Venteicher et al. (PDF 49.5 kb) [file 12885_2018_4251_MOESM8_ESM.pdf]

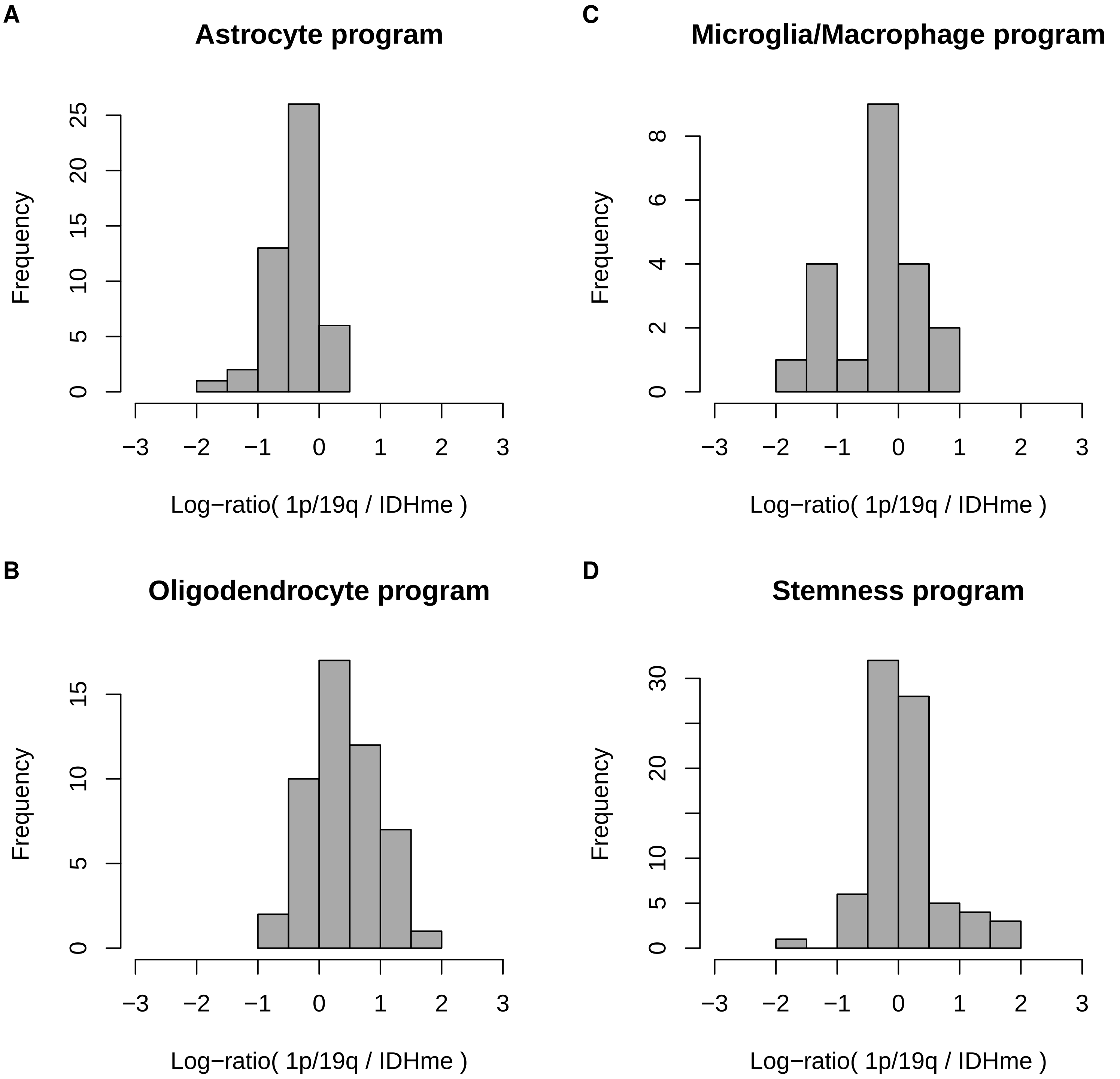

Figure S1: Comparison of 1p/19q and IDHme to characteristic expression programs from Venteicher *et al.* (2017). Genes included in specific expression programs were taken from Table S3 of Venteicher *et al.* (2017). We utilized these genes and determined their average $\log_2$-ratios for tumors of the 1p/19q subgroup vs. tumors of the IDHme subgroup and plotted corresponding histograms. **(A)** Genes of the astrocyte-like program tend to show lower expression levels in 1p/19q than in IDHme. **(B)** Genes of the oligodendrocyte-like program tend to show greater expression levels in 1p/19q compared to IDHme. Means of (A) and (B) are significantly different (P = 4.8e-11). **(C)** Differences in the expression of microglia/macrophage markers between 1p/19q and IDHme. Mean is significantly different from zero (P < 0.03). **(D)** Majority of genes of the stemness program do not differ in their expression between 1p/19q and IDHme, except for several strong outliers. Mean is not significantly different from zero (P = 0.12).
